# Influence of Process Parameters on the Porosity, Accuracy, Roughness, and Support Structures of Hastelloy X Produced by Laser Powder Bed Fusion

**DOI:** 10.3390/ma12193178

**Published:** 2019-09-27

**Authors:** Flaviana Calignano, Paolo Minetola

**Affiliations:** Dipartimento di Ingegneria Gestionale e della Produzione, Politecnico di Torino, Corso Duca degli Abruzzi, 24, 10129 Turin, Italy; paolo.minetola@polito.it

**Keywords:** Hastelloy X, nickel super-alloys, selective laser melting, additive manufacturing, process parameters

## Abstract

The manufacture of highly complex components from nickel-based superalloys with laser powder bed fusion (L-PBF) technology can reduce the production costs parts with comparable microstructural and mechanical properties when compared to casting. The purpose of this study was to investigate the characteristics of samples produced in commercial Hastelloy X (with w% composition of 21Cr-18Fe-9Mo-0.7W-1.5Co-0.1C-1Si-1Mn-0.5Al-0.15Ti-bal.Ni) with an L-PBF process in terms of build density, accuracy, surface roughness, and interface area between the part and the support structures. Samples were obtained with a high density (99.88%), without cracks and with low surface roughness. From the analysis of the support structures, it emerged that the choice of the parameters between support structures, the lower face of the part (down-skin) and the internal area of the part (in-skin) is fundamental to the correct realization of these structures in order to avoid deformation of the components that is induced by thermal stresses during part building.

## 1. Introduction

**Figure 1:**
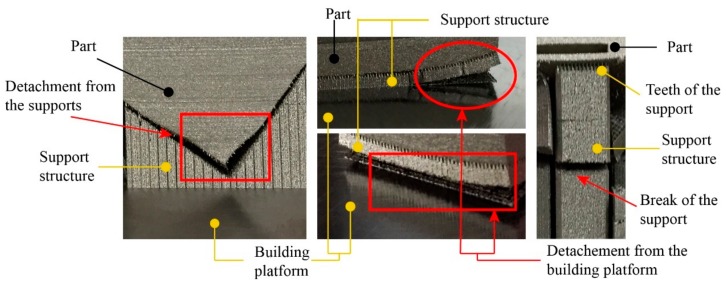
Detachment of the supports from the part (**left**), distortion caused by the detachment of the supports anchored to the part by the building platform (**center**), and breaking of the support (**right**) during the manufacturing of a component in nickel alloy 718.

Laser powder bed fusion (L-PBF) is an additive manufacturing (AM) process, which enables manufacturing of lightweight and complex metallic structures according to a CAD (computer aided-design) model utilizing a high-energy focused laser beam to selectively melt powdered metal material layer-by-layer. This process has great potential for the fabrication of highly complex high-strength and high-temperature components from nickel superalloys with microstructure and mechanical properties comparable or superior to casting and forging processes [1]. The characteristic properties of superalloys, such as increased hardness and tensile strength, can make them difficult to form using conventional techniques [2]. For this reason, in recent years, the manufacture of complex full density superalloy components through L-PBF has become an area of great interest. Ni-based superalloys are widely used in the aerospace [3], marine [4], nuclear reactor [5], and chemical industries [6] due to their superior mechanical and chemical properties. One of the main issues encountered in L-PBF of nickel-based superalloys components such as Hastelloy X and Inconel 738 [7,8,9], is the sensitivity to the formation of micro-cracking. Controlling laser scanning parameters and heated platforms [1,2,10,11] can reduce micro-cracking and thermal stresses. Generally, in order to consolidate the cracks, Hot Isostatic Pressing (HIP) is used. However, surface cracks and open porosity remain [1], and the HIP process leads to coarsening of grains [10]. For these reasons, some researchers have highlighted that the laser parameters control and/or post-processing cannot be considered definitive solutions, and a more effective solution may require the optimization of the alloy during processing. Some studies have moved in this direction by analyzing, for example, the effects of minor elements, specifically Si and Mn, on the crack susceptibility of Hastelloy X during L-PBF processing [2]. The hypothesis was based on the idea that the micro-segregation of those elements towards the grain boundaries was giving rise to weakened brittle phases, thus increasing the possibility of micro-cracking. This effect has been compared with the findings of Savage and Krantz [11], thus proving to be an important factor in the cracking of autogenous welds of Hastelloy X. Although, in the literature, there are various studies aimed at producing dense Hastelloy X samples, up to date, according to the authors’ knowledge, there are no studies focused on the analysis of support structures for this alloy. In practice, the freedom of design is limited due to the presence of support structures, which are necessary above all when the amount of overhanging surfaces exceeds a certain threshold value which generally depends on the material. The function of the support structures is also to conduct the excess heat produced by this process to avoid large temperature differences and thermal stresses. A large temperature difference can cause warping and/or collapse of the part. Furthermore, the support structures can fix the product to the building platform and resist the residual stresses and forces of the recoating blade. The blade pushes a quantity of powder which can cause dynamic pressures against the leading edge of the part under construction. Some researches focused on reducing the number of required support structures by orientating the object into an optimal building position. Calignano [12] researched the manufacturability of overhanging structures using optimized support parts for aluminum and titanium alloys. Hussein et al. [13] described the new application of lattice structures with very low volume fraction for lightweight support structures. Their study has showed that the type of support, the fraction of volume, and the size of the cells are the main factors that influence the producibility, the amount of support, and the build time of the lattice support structures. Titanium and nickel alloys are more susceptible to detachment from supports compared to aluminum. The detachment of the supports from the part side or even from the platform (Figure 1) can take place not only for a non-optimal design of the supports but also for a wrong choice of process parameters. Thermal stresses can cause huge deformations to the part and can lead to process failure due to delamination of the part from the building platform. In some cases, they are so high that they can also induce cracks in the parts during processing before the build is completed. The difference of the laser power between the last layer of the support (teeth [12]) and the first layer of the part (down-skin) creates a narrowing of the melting region with consequent deformation and detachment of the part from the supports.

In this study, the interface between the part and the support structures of samples produced in Hastelloy X was analyzed by investigating different process parameters. These have been varied both for the part, in order to obtain a dense sample, and for the support structures. Furthermore, the dimensional accuracy and surface roughness of the samples were analyzed in order to have a complete view of the optimal process parameters for this alloy used in an L-PBF system with a laser power of 200W. 

## 2. Materials and Methods 

Commercially available gas-atomized Hastelloy X powder provided by EOS GmbH, with particle sizes ranging from 23 to 63 μm and w% composition of 22Cr-18Fe-9Mo-1.5Co-0.6W-0.1C-1Si-1Mn-0.5Al-0.15Ti-bal.Ni was processed using an EOSINT M270 Dual-mode system equipped with an Yb-fiber laser. The maximum laser power is equal to 200 W and the beam-spot size is 100 μm. The building platform is heated at 80 °C to reduce thermal stresses that arise during the process. Cubic samples of 10 mm × 10 mm × 10 mm were produced under Ar atmosphere (oxygen content lower than 0.10%) and analyzed in the as-built condition. The process parameters used are shown in Table 1. Three replicas of each sample have been produced.

**Figure 2:**
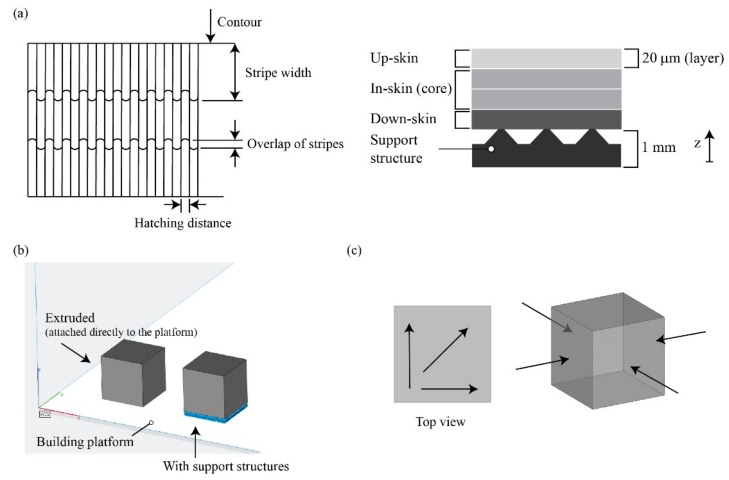
(**a**) Schematic of process parameters; (**b**) manufacturing strategy; (**c**) direction of roughness measurements.

In general, a hatch operation is used to create the volume of the structure, while a contour operation is used to improve dimensional accuracy and improve the surface finish of the final structures. For each scanning method, there are also several parameters: laser power, scan speed, beam offset, overhanging parameter adjustment, skin parameter adjustment, hatch pattern adjustment, and cleaning gas flow [14]. Among these parameters, laser power, scan speed, and hatching distance directly control the input energy density and thus the amount of material melted. The beam offset compensates for the potential geometrical error caused by the characteristic energy beam size. The hatching area is composed of three sections called in-skin (or core), up-skin (located on the upward surfaces of the part), and down-skin (located on the downward surfaces of the part). In this study, first the samples have been produced with the same parameters of laser power (*P* (W)), scanning speed (*v* (mm/s)), and hatching distance (*h_d_* (mm)) for the three zones and built attached to the building platform (extruded). Process parameters as stripe width (5 mm), overlap of stripes (0.12 mm), layer thickness (20 µm), and contour (*P* of 150 W and *v* of 1250 mm/s) were kept constant (Figure 2a). The scanning path of the laser beam is rotated by 67° with respect to the previous layer to guarantee a high final density and isotropic properties on the building plane [15]. The porosity, accuracy, and roughness were calculated on these first samples. Then, the best parameters of density and roughness were chosen to build the samples with support structures (Figure 2b) in order to investigate the suitable parameters to avoid the detachment of the part from the supports used in the L-PBF process [12,13,16]. The height of the support structures was 1 mm. The density of the samples was measured by the Archimedes method. The dimensional accuracy was measured by means of a micrometer. For each sample, five different locations were considered. The surface roughness of the sample was measured as-built with the use of an RTP80 roughness tester by SM Instrument to obtain the values of roughness average *R_a_*, and average maximum height of the profile *R_z_*. The measuring distance was 4.8 mm and a cut-off filter of 0.8 mm was used. Surface roughness was measured in the top and lateral areas. The roughness measurement was performed on the samples taking three measurements on the upper face in three different directions and three measures on the side faces in four different directions (Figure 2c). The samples were investigated by optical microscopy (Leica EZ4D stereomicroscope). The samples for optical observation were prepared by grinding surfaces up to 2400 SiC papers.

## 3. Results and Discussion

### 3.1. Porosity

**Figure 3:**
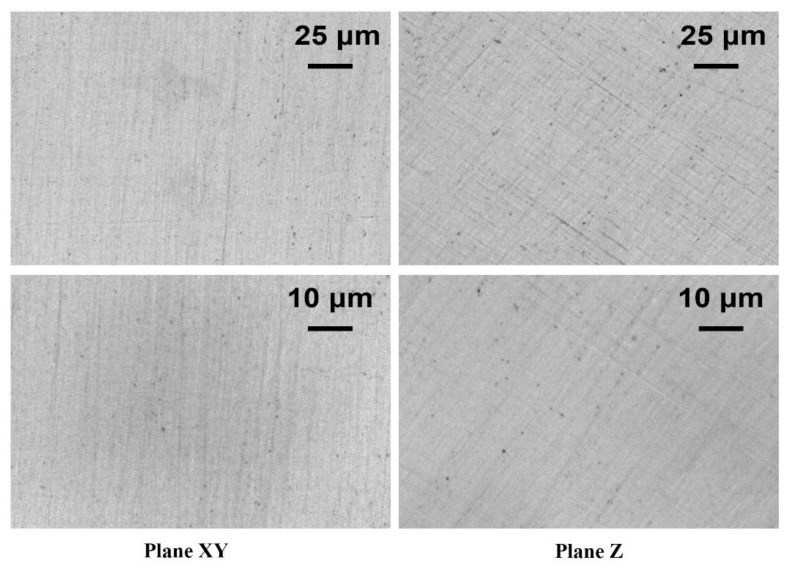
Optical images of the sample produced with laser power of 195 W, scan speed of 1000 mm/s, and hatching distance of 0.05 mm/s.

The minimum porosity obtained for the samples produced was 0.065 ± 0.032% for *P* of 195 W, *v* of 1000 mm/s and *h_d_* of 0.05 mm/s. The values obtained are comparable with those found in literature at the density level [8,17,18,19,20] but, unlike the samples obtained from other studies carried out with laser powers of 200 W [18,19], the sample did not show any cracks (Figure 3). 

**Figure 4:**
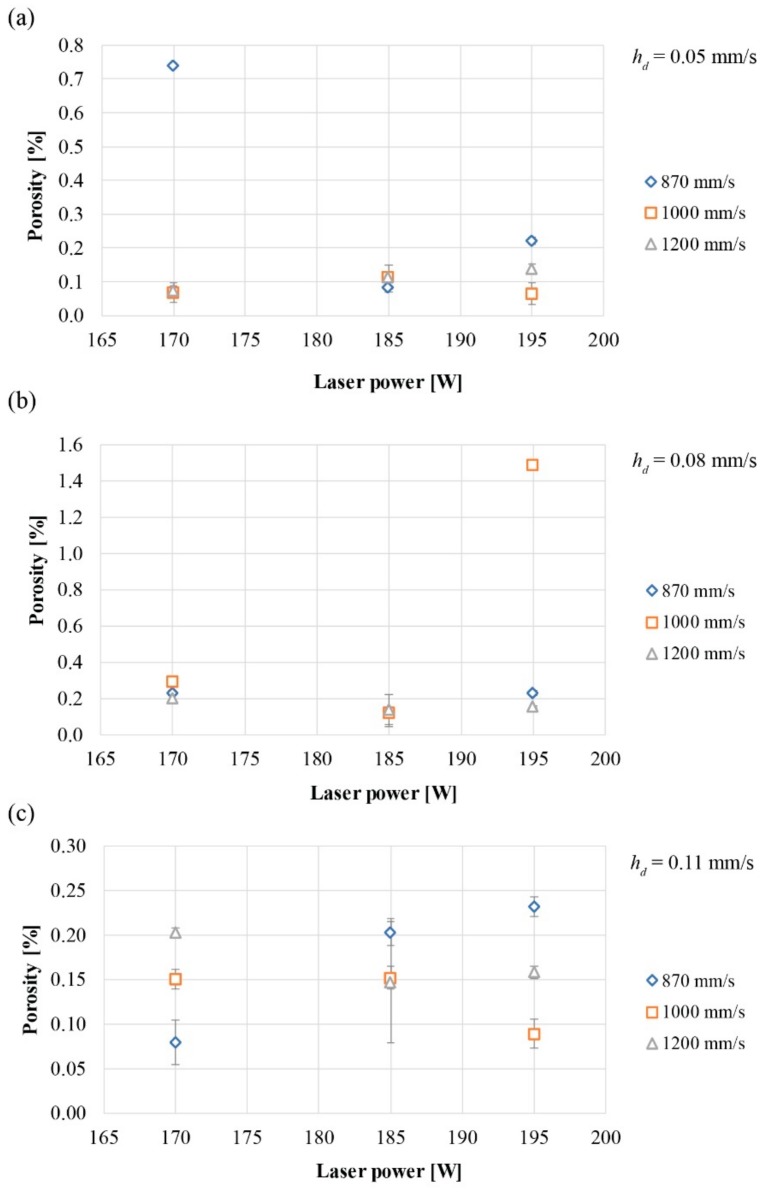
Porosity obtained for different values of scan speed and laser power with respect to hatching distance of (**a**) 0.05 mm/s, (**b**) 0.08 mm/s, and (**c**) 0.11 mm/s.

Comparing the results with respect to *h_d_* (Figure 4), the greatest variability is found for a *h_d_* of 0.11 mm/s. In particular, it is possible to see that the porosity increases with increasing *v* for *P* less than 185 W, while it decreases if the *P* increases with increasing *v*. For the *h_d_* of 0.05 mm/s, the porosity is higher using *P* and lower *v*. As the *v* increases with the same *P* of 170 W, the porosity is considerably reduced: From 0.74 ± 0.008% to 0.068 ± 0.028% for *v* of 1000 mm/s and 0.075 ± 0.012% for *v* of 1200 mm/s. For an *h_d_* of 0.08 mm/s, the lower porosity is obtained for a *P* of 185 W regardless of the *v* used: 0.131 ± 0.009%, 0.123 ± 0.021%, 0.139 ± 0.082% for *v* of 870 mm/s, 1000 mm/s, and 1200 mm/s, respectively. For the *h_d_* of 0.11 mm/s, the porosity is reduced for low *P* and low *v* and for high *P* and *v* of 1000 mm/s.

### 3.2. Accuracy

**Figure 5:**
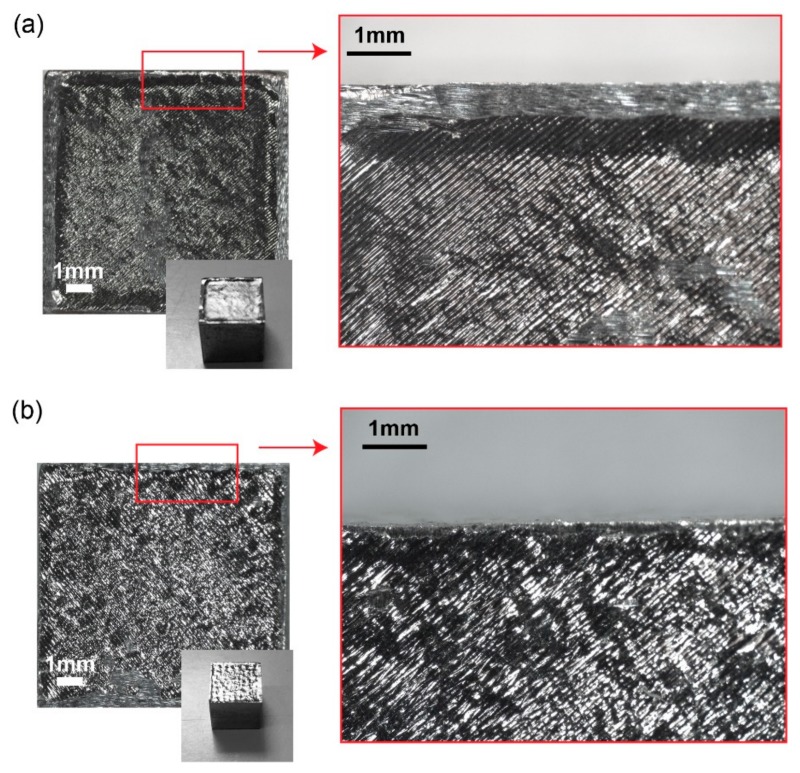
(**a**) Sample with inaccuracy on the up-skin surface: The sample has the contour raised above the internal area (up-skin); (**b**) sample without defect on the up-skin surface.

The measured dimensions differ from the CAD model of about 10.06 mm ± 0.02 mm. Looking at some samples, an upward edge effect was noticed in the last layer (up-skin) (Figure 5). There exist two compensation parameters of the beam offset: The laser beam size offset (BO_1_) and the user defined in-process beam offset (BO_2_). BO_1_ is determined by the laser optics and material powder characterization. BO_2_ is associated with the custom adjustment necessary for different geometries and energy requirements. Therefore, BO_2_ could vary with each design. In the hatch operation, the total offset determines the distance between the outer boundary of the scan pattern and the contour of the CAD model. The total offset value is determined by the sum of the two offset values. For the contour operation, the same rule of calculating the total offset is applied. For the Hastelloy X, the BO_1_ and BO_2_ of the nickel alloys was used, that is 0.064 mm and BO_2_ of 0.012 mm for the contour. Since the problem occurred only for some samples, in particular those produced with *v* of 870 mm/s and *h_d_* of 0.05 mm, the problem may not be due to the choice of BO parameters but to residual stress [21]. In the L-PBF process, the main source of the residual stresses is caused by the rapid heating and cooling cycles of successive layers that generate large thermal gradients. Due to thermal expansion, the top of the layer (exposed to the laser) experiences a tensile stress, while the cooler interface (interface between the current layer and the previous one) has compressive stresses acting on it. The problem occurs mainly when the underlying layers limit the thermal expansion and contraction of the layers immediately below the melt pool. This can cause an elastic compressive strain within the layers, resulting in a stress gradient between the layers. The combination of some filling parameters with those of the contour has led to an excess of stress with the consequent effect of raising the edges.

### 3.3. Surface Roughness

**Figure 6:**
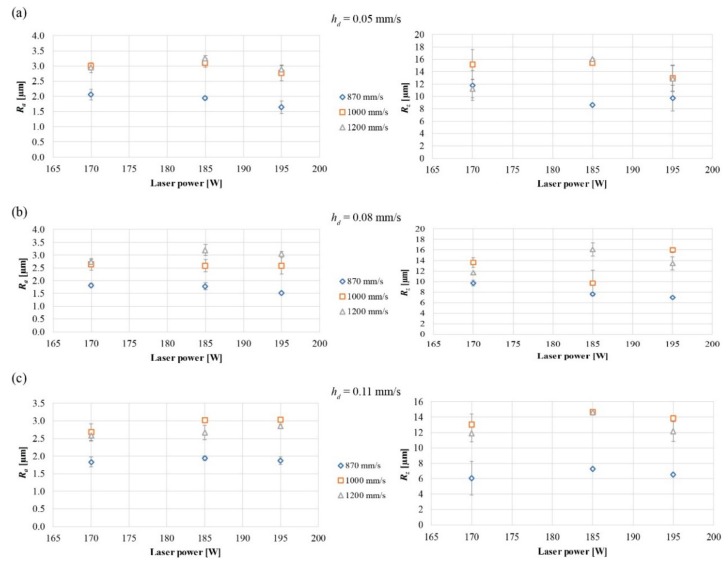
Values of surface roughness *R_a_* (**left**) and *R_z_* (**right**) measured in the top areas for different values of scan speed and laser power with respect to hatching distance of (**a**) 0.05 mm/s, (**b**) 0.08 mm/s, and (**c**) 0.11 mm/s.

Figure 6 shows the average values of the measurements taken on the top of the samples. As can be seen from the value of the standard deviation, the average value is able to correctly represent the roughness values (*R_a_* and *R_z_*) reducing to the minimum the error induced by the calculation of the average. The sample produced with *P* of 195 W, *v* of 870 mm/s, and *h_d_* of 0.08 mm/s has the least surface roughness (*R_a_* of 1.51 ± 0.01 µm and *R_z_* of 6.98 ± 0.04 µm). If the results on the top area are compared with those in the literature, the values obtained in this study are much lower (7–30 µm [22], 7 µm [8]). The surface roughness on the contour has a minimum value of *R_a_* of 10.25 ± 0.32 µm and *R_z_* of 54.71 ± 0.03 µm for *P* of 195 W, *v* of 1000 mm/s, and *h_d_* of 0.05 mm, while it has a maximum value of *R_a_* of 18.57 ± 0.30 µm and *R_z_* of 83.53 ± 3.12 µm for *P* of 195 W, *v* of 870 mm/s, and *h_d_* of 0.05 mm. Despite having used the same parameters, the variation of the values of the walls’ roughness can be singularly attributed to two factors typical of the L-PBF process or to their combination: Spatters and surrounding powder. Liquid spatters formed during the interaction between laser–powder–melt pool can tend to contaminate powder beds and parts built nearby. The samples are constructed in a bed of powder and part of the heat released during the construction of these is carried on the powder bed causing the adjacent particles to reach partial melting/sintering points and thus to stick to the surface of the sample [23].

### 3.4. Support Structures

Having identified the best parameters in terms of density and surface roughness, these have been used to construct the samples with the support structures. During the production of the samples, after the construction of the supports and the first four layers of the samples, the processing (called “job” in technical term) had to be interrupted due to the delamination of the subsequent layers. Observing the layers, a problem was noted concerning not the choice of the type of support but of the process parameters. The choice of these for the zones between support down- and in-skin can influence the construction of the part. Therefore, it was decided to modify the down-skin parameters using *P* of 80 W and *v* of 400 mm/s for the supports since these showed a better adhesion with the building platform. Table 2 shows the process parameters used for down-skin, the energy density (*E_d_* = *P*/(*v* × *h* × *t*)) and linear energy (*E_l_* = *P*/*v*) [24]. For Sample 1, the parameters previously selected as best for density were used in order to have a comparison sample.

**Figure 7:**
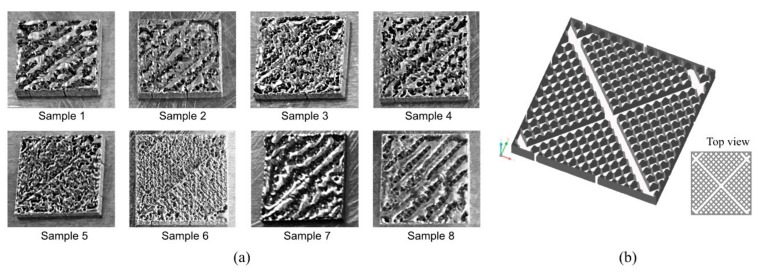
(**a**) Interface between support structures, the lower face of the part (down-skin), and the internal area of the part (in-skin); (**b**) support structure of block type.

Sample 6 shows a fused area with the previous layers that led to the construction of samples without problems. The other samples show some balling problems which caused a deformation of the part that led to the collision of the sample with the blade and subsequent delamination of the part. This could be attributed to the relatively lower heat transfer into the powder for support as opposed to the extruded sample. The supports are generally designed as thin structures with high porosity to facilitate their removal while reducing the construction time and the amount of powder needed to make them. Nadammal et al. [25] have shown that the heat transfer rates dictated by the supports configurations along with the scanning strategy influence the development of residual stress. The fabrication of supports leaves residual powder trapped inside the channels (Figure 7b). Therefore, the first layers of the part are built on a mixture of powder and thin walls. In the area of contact between the support and the construction platform, heat can be transported rapidly to the substrate because of the greater thermal conductivity of the solid part. As the number of layers increases and therefore the distance from the substrate increases, the heat in the melt pool cannot be dissipated over time because the actual conductivity of the powder bed is much lower.

Furthermore, by observing the energy density of the various samples, Sample 6 was built with lower energy density and is less affected by the lower heat transfer due to the powder. As the height of construction increases, the core part of the samples becomes independent of the support structures and the samples show the same characteristics as those grown-ups attached to the building platform. An increase in the volume fraction of the solid material, i.e., the number of cells in the support structure with corresponding volume drift of trapped powder, would allow increasing the effective thermal conductivity by reducing the problems during the construction of the part. However, this solution would increase the construction time and the difficulty of removing the supports. Observing the values of linear energy (Table 2), it can be noted that the ratio between *P* and *v* is not able to explain the phenomenon as samples with the same *E_l_* value has shown a different behavior. From this result, it can be stated that the hatching distance has a certain relevance in combination with other parameters [26,27].

## 4. Conclusions

In this research, the effects of processing parameters (laser power, scan speed, hatching distance) on the density, accuracy, surface roughness, and support structure of the Hastelloy X produced by L-PBF process have been experimentally studied in order to get the complete set of technological parameters for the layerwise production of components using this alloy. It was found that the density is high (99.88%) with a layer thickness of 20 µm, when the laser power is 195 W, the scan speed is 1000 mm/s and the hatching distance is 0.05 mm. In the as-built samples, cracks were not found. Furthermore, the samples have an accuracy that falls within the general tolerances of the L-PBF process. The obtained surface roughness is low compared to the values present in the literature: For the top surfaces, a minimum of nearly 1.51 µm *R_a,top_* and a maximum of 3 µm *R_a,top_*; while for the side surfaces, a minimum of nearly 10.25 µm *R_a,contour_* and a maximum of 18.57 µm *R_a,contour_*. 

Besides influencing the success or failure of the fabrication of a part, the support structure can influence the residual stress. Therefore, a right combination of support structure type and process parameters can help to overcome residual stress-induced part distortion in the areas of interaction between support and part, and to optimally conduct excess heat away from the part. In this study, it was found that the combination of *P* of 80 W and *v* of 400 mm/s for the supports, *P* of 170 W, *v* of 1000 mm/s, and *h_d_* of 0.08 mm for down-skin led to the construction of samples without problems.

## Figures and Tables

**Table 1:** Process parameters.

	*P* (W)	*v* (mm/s)	*h_d_* (mm)
In-skin/up-skin/down-skin	170, 185, 195	870, 1000, 1200	0.05, 0.08, 0.11
Supports	80, 90	400, 500	

**Table 2:** Process parameters, energy density and linear for down-skin and support.

	Sample	*P* (W)	*v* (mm/s)	*h_d_* (mm)	*E_d_* (J/mm^3^)	*E_l_* (J/mm)
Down-skin (2 layers)	1	195	1000	0.05	195.00	0.195
	2	170	1000	0.05	170.00	0.170
	3	195	1000	0.08	121.88	0.195
	4	195	870	0.08	140.09	0.224
	5	170	870	0.08	122.13	0.195
	6	170	1000	0.08	106.25	0.170
	7	170	870	0.05	195.40	0.195
	8	195	870	0.05	224.14	0.224
Supports	-	80	400	-	-	0.200
